# The management of anticoagulated fragility femoral fracture patients

**DOI:** 10.1177/11207000241282303

**Published:** 2024-09-23

**Authors:** Muhamed M Farhan-Alanie, William G P Eardley

**Affiliations:** 1 Warwick Medical School, University of Warwick, Coventry, UK; 2 South Tees Hospitals NHS Foundation Trust, Middlesbrough, UK; 3 University of Teesside, Middlesbrough, UK; 4 University of York, UK

**Keywords:** Anticoagulation, delay, femur, fracture, fragility, surgery

## Abstract

Approximately 20% of patients sustaining a fragility femur fracture use an anticoagulant, and over 30% use an antiplatelet medication, both of which can result in surgical delay. Previously confined to fractures of the proximal femur, performance assessment, outcome and surgical delay is now assessed for all fractures of the femur in older patients, including those involving implants. This narrative review draws together all literature pertaining to anticoagulation and antiplatelet management in older patients with a fracture of the femur to address 5 key points: prevalence of anticoagulant and antiplatelet use; analysis of management protocols; collation of national guidelines; comparison of perioperative management; timing of surgery and perioperative outcomes.

Our review found that the prevalence of fragility femur fracture patients taking anticoagulant and antiplatelet medication ranges from 20–40% and 25–35% respectively. More anticoagulated patients are taking direct oral anticoagulants compared to vitamin k antagonists with growing implications for variation in practice and delays to surgery.

Several national guidelines exist although these are characterised by marked variation, there is little standardisation, and none are generalised across all fragility femur fractures.

Expedited surgery within 36 hours of admission in patients taking an anticoagulant or antiplatelet medication is safe and has been demonstrated in fractures of the proximal femur across many small number studies although no such evidence exists in non-proximal femur fractures despite this population sharing similar characteristics. There is a need for all fractures of the femur in older people to be considered when researching and assessing performance in this population to prevent needless variation and delay.

## Introduction

Around 75,000 older people have a low-energy fracture of a part of their femur every year in England and Wales alone.^1^ It is the commonest reason for older people to have an emergency operation and its incidence increases.^2,3^ Although most have sustained a proximal femur (hip) injury, those fractured in other parts of the femur or around existing implants in the bone are equally important, share comparable characteristics and require similar complex multidisciplinary care assessments and pathways.^4,5^ Consequently, these patient groups can be referred to and grouped together using a broader and more encompassing term of “fragility femur fracture patient”. This perspective has recently been officially adopted by the National Hip Fracture Database of England and Wales (NHFD) which has adapted by expanding collection of data on performance metrics for fractures involving the whole femur including periprosthetic fractures.^6^ These changes reflect the progression towards the inclusive concept of the fragility femur fracture and the recognition of frailty in this broader injury population.^7^

Within this large group of patients, a common issue is the perioperative management of anticoagulation medications.^8,9^ Wide variation in the standard of care of patients taking oral anticoagulation medications such as warfarin (Vitamin K Antagonist [VKA]) and direct oral anti-coagulants (DOAC) exists.^10^ Similarly, albeit to a lesser extent, this issue also affects patient taking antiplatelet medications such as aspirin and clopidogrel.^11,12^ The magnitude of this problem has grown as the proportion of patients using these medications has increased. Approximately 20% and 30% of the total injury group use an anticoagulant and antiplatelet medication, respectively.^8,9,13–15^ This situation has arisen because of increased life expectancy and a growing burden of multimorbidity associated with polypharmacy.^16–18^

Guidelines from the National Institute for Health and Care Excellence (NICE) and the British Orthopaedic Association (BOA) advise surgery to be performed no later than the day after, and within 36 hours of, the patient’s admission respectively.^7,19^ Specific guidance on the management of anticoagulated patients is also included within these guidelines. NICE guidelines state that anticoagulated patients should be optimised preoperatively to prevent their surgery being delayed while the British Orthopaedic Association guidelines state that protocols for anticoagulation reversal must be available.^7,19^ Despite this, several small studies suggest that disparity exists among femoral fragility fracture patients taking anticoagulants and antiplatelet medications, and is associated with delayed surgery and increased mortality.^9,10,15,20–22^

This narrative review draws together all literature pertaining to anticoagulation management in older patients with a fracture of the femur to address 5 key points:

- Prevalence of anticoagulant and antiplatelet use
- Analysis of management protocols
- Collation of national guidelines
- Comparison of perioperative management e.g. reversal
- Timing of surgery and perioperative outcomes

## Prevalence of anticoagulant and antiplatelet use

VKAs and DOACs are the main anticoagulant medications taken by patients admitted to hospital with a fragility femoral fracture. In the UK, the prevalence of such patients using either anticoagulant type is around 20%.^8,9^ Of importance however, these figures are based on data from study periods ranging from 2014 to 2019 only and therefore, are likely an underestimate. As perspective, for fragility femur fracture patients in other parts of the world, the percentage of these medications is reported as up to 40%.^13,23,24^ Although DOAC medications were introduced to the UK much later than VKA, recent studies show that the majority of anticoagulated femoral fracture patients are now using these medications.^23,25,26^ The relative proportion of DOAC users is predicted to increase further over time due to the reduced prescription of VKA.^27^ This is likely multifactorial of warfarin’s extensive interactions and narrow therapeutic range requiring regular monitoring of serum International Normalised Ratio (INR).^28^ Another reason may be the increased femoral fracture risk shown to be associated with warfarin use.^29^ Furthermore, despite stringent monitoring, warfarinised patients spend only 55% of their time within their therapeutic range.^30^ As such, it is not uncommon that fragility femur fracture patients present with INR levels substantially higher than their desired therapeutic range on presentation,^31^ which potentially complicates reversal prior to surgery even further.

Fortunately, this is a scenario which is becoming increasingly less common over time due to a diminishing prevalence of patients being prescribed VKA. However, in exchange, a new problem presents with DOAC medications in that they are not easily reversed though clearance of the drug is relatively quicker than VKA and more predictable.^32^ Although it is possible to measure DOAC plasma levels, this is not routinely performed by many units due to its predictable clearance, rendering this investigation of unclear value in clinical practice.^33,34^ In addition, a recent study comparing the effects of four different ranges of DOAC plasma levels (<30 ng/ml, ⩾30-49 ng/ml, ⩾50–79 ng/ml, ⩾80 ng/ml) in femoral fracture patients found no significant differences in blood loss between the groups.^33^

With regard to antiplatelet medications, a relatively greater proportion of femoral fragility fracture patients take these agents. Current prevalence in the UK is unknown although data from the most recent study on this topic completed 5 years ago reports a prevalence of 25%.^9^ This figure reaches 30% or higher in some studies completed abroad including Australia and Japan.^15,35^ It is important to mention there is also a small subgroup of patients who take both antiplatelet and anticoagulant medications however their current prevalence is less well defined in the literature.

## Analysis of management protocols

Many protocols aiming to improve time to surgery in anticoagulated fragility femur fracture patients taking warfarin and DOAC medications have been developed.^36,37^ However, substantial variation exists between available protocols for both types of these anticoagulant medications.^38^ For VKA reversal they vary in terms of need for reversal agent, type of reversal agent, timing of administration, route and dose. Most of these protocols advise administration of vitamin K in isolation however there are also a few which advise the use of prothrombin complex concentrate (PCC) in conjunction with vitamin K, and fresh frozen plasma (FFP) on its own. Although these reversal agents are all associated with risks of harm to patients, vitamin K is relatively safer than both FFP and PCC.

Reported risks of vitamin K use include thrombosis, warfarin resistance on restarting, and anaphylaxis.^39,40^ Incidence of the latter has been reported to be 3 cases per 10,000 doses with intravenous (IV) vitamin K. Complications are more common with FFP at an estimated incidence of 1 case per 2184 transfusion.^39,41^ More concerning is the 15% risk of thromboembolic events when PCC is used.^42^ This risk with FFP is approximately 3% however administration also carries the risk of precipitating fluid overload which anticoagulated patients may be at particular risk of, given a common indication for these medications is atrial fibrillation which often coexists with ischaemic heart disease.^43^ In addition to risks to patient care, there are also relatively greater cost implications of using FFP and PCC.^44^

Similar protocols for DOACs and antiplatelets also seem to vary with regards to length of time delay prior to surgery and whether this is required. Some DOAC protocols also specify restrictions based on the different medications available.^38^ In 2007, 2 UK national surveys were completed investigating clinicians’ management of hip fracture patients taking antiplatelets, and demonstrated wide variation between hospital protocols and practice. Approximately 21% and 14% of units delayed surgery for more than 5 and 7 days following discontinuation of clopidogrel.^11,12^ A more recent survey on this topic was completed in 2015 in the Unites States, and revealed that approximately 30% of surgeon respondents would opt for delaying surgery in patients with hip and distal femur fractures while approximately 21% would delay for a femoral shaft fracture.^45^

Despite these variations, the presence of protocols on the whole have been shown to be effective in improving time to surgery with no differences in adverse events. A meta-analysis evaluating the effect of the application of such protocols demonstrated a significant mean reduction in time from admission to surgery of 45.31 hours (95% confidence interval [CI],15.81–74.80; *p* = 0.003)^38^ (*n* = 427 patients; 6 studies) in patients taking VKA medications. Similar analyses for patients using DOAC and antiplatelet medications were not possible to perform as there have not been any completed studies on this specific topic. The authors also investigated other outcomes in their meta-analysis including rate of blood transfusion and found there were no differences between the between patients who followed a VKA-reversal protocol and non-anticoagulated control patients (odds ratio [OR] 1.08; 95% CI, 0.53–2.20; *p* = 0.08) (*n* = 771 patients; 3 studies). A comparison of blood transfusions within 48 hours postoperatively between non-anticoagulated patients and those using DOAC medications was also carried out. This demonstrated a significantly higher rate of blood transfusion among DOAC-treated patients (OR 0.58; 95% CI, 0.36–0.96; *p* = 0.03) (*n* = 1958 patients; 4 studies). However, it is worth highlighting that 1 of the 4 studies included in this analysis investigated patients who only received intramedullary nail fixation,^14^ and this was the only study which demonstrated significant differences on the forest plot. It has been established that different surgical procedures for fragility femur fracture patients have differing associated risks of blood transfusion, and the results of this single study may have skewed the meta-analysis results for this outcome.^46^ It is also worth mentioning there were differences in the types of procedures performed between the patient groups in the other studies. Separate meta-analyses stratified by type of procedure would have helped allow greater interpretation of the results. However, this may not have been possible due to composite reporting of results within the individual studies. Of note, other studies controlling for type of procedure and other confounding factors have demonstrated no difference in change in haemoglobin value and blood transfusion requirements in patients taking DOAC medications.^47^

It is also important to consider that investigating blood transfusion may be influenced by the initial trauma which has been shown to be associated with a larger drop in haemoglobin than the operation itself.^48^ Isolating the effect of the surgery on transfusion requirements in anticoagulated patients is therefore challenging. Differences may be over-estimated when comparing to a patient group which was optimally anticoagulated at the time of injury. This is supported by the findings of the study by Hofer et al.^33^ which found a significant association between low haemoglobin on admission and patients with highest DOAC plasma level (⩾80 ng/ml). There are other confounders to consider including the significantly higher level of comorbidity of anticoagulated fragility femur fracture patients which is associated with lower serum haemoglobin levels (*p* < 0.01).^10,49^ Lastly, despite the slightly higher blood transfusion requirement in patients taking DOAC medications identified in the meta-analysis by You et al.^38^ the 30-day mortality was similar between patient groups (OR 1.30; 95% CI, 0.49–3.43; *p* = 0.6) (*n* = 1775 patients; 3 studies) suggesting surgery in anticoagulated patients within 48 hours of presentation was safe.^38^

## Collation of national guidelines

The Association of Anaesthetists of Great Britain and Ireland (AAGBI) and British Committee of Standards for Haematology (BCSH) guide the management of patients on anticoagulation and antiplatelet therapy requiring surgery.^26,50^ NHS Scotland have also produced separate guidelines on the management of femoral fracture patients on anticoagulants and antiplatelets.^51^

The AAGBI guidelines which are endorsed by the British Geriatric Society (BGS) focus predominantly on fragility femur fracture patients. For patients taking VKA medications, this recommends checking INR levels and administering 5 mg IV vitamin K as soon as possible in the Emergency Department. Where the INR level returns >1.5 after 4–6 hours, it is advised that a repeat dose of vitamin K or alternatively PCC should be given. However, the recommended threshold INR level for proceeding with surgery is reported to be 1.8 or lower. If neuraxial anaesthesia is preferred then the recommended threshold INR level is 1.5.^26^ For DOAC medications, the guidelines advise surgery to be delayed for the equivalent of 2 or 4 times the half-life of the medication since last dose depending on whether creatinine clearance is above or below 30 ml/minute for Factor Xa inhibitors (apixaban, edoxaban, rivaroxaban). This results in approximately 25% residual anticoagulant effect of medication remaining in the patient’s system and is thought to provide an appropriate compromise between the competing risks of harm versus benefit.^26^ It is also advised that plasma DOAC level is tested in patients with poor renal function and advises to proceed with surgery if the result is <50 ng/ml. For dabigatran, which is a thrombin inhibitor, the guidelines suggest planning surgery on the afternoon of the day after admission and measuring thrombin time in the morning of this day. The guidelines recommend seeking haematology advice where the thrombin time returns abnormally prolonged. For patients taking aspirin or clopidogrel, the guidelines recommend proceeding with surgery and considering platelet transfusion where there are concerns regarding bleeding for patients using the latter.

More generic guidelines on the perioperative management of patients on anticoagulation have been published by the BCSH however these are not specific to the fragility femur fracture population and respective procedures performed.^50^ This may be partly the reason why the recommendations slightly differ to those of the AAGBI guidelines. Although the BCSH guidelines also recommend 5 mg IV vitamin K where surgery can be delayed for 6–8 hours time, a relatively lower INR value of 1.5 is advised prior to proceeding with surgery compared to the AAGBI guidelines.^52^ These guidelines also consider the different DOAC medications and patient’s renal function for recommendation on duration of length of time these should be discontinued prior to surgery. Broadly speaking the guidelines advise discontinuation of the DOAC medication for 24–72 hours or up to 96 hours depending on renal function and whether the procedure is deemed low or high risk by the surgeon. However, assessment of this risk is not defined within these guidelines though previous studies have classified femoral fracture surgery as being high risk for bleeding.^53^ The applicability of this guideline to different patient populations and surgical procedures is important to consider particularly as the recommended durations in this document would result in delays associated with significantly increased harm observed in femoral fracture patients.^54^ The BCSH guidelines also suggest the use of tranexamic acid where an anticoagulant effect may persist. In unselected fragility femur fracture patients, this has been shown to be effective in reducing blood transfusion (relative risk [RR] 0.83; 95% CI, 0.75–0.91; *p* = 0.001) with no differences in 30 and 90 day mortality in femoral fracture patients (RR 0.84; 95% CI, 0.65–1.08; *p* = 0.175, and RR 1.24; 95% CI, 0.93–1.66; *p* = 0.141 respectively).^13^ This effect may be relatively greater in anticoagulated patients however, no sub analysis was performed to isolate the effect estimate for this patient group. In contrast, the AAGBI guidelines advocate tranexamic acid use according to individual hospital protocols.

NHS Scotland in collaboration with the Scottish Hip Fracture Audit have produced a detailed and specific summary on the management of patients on anticoagulants and antiplatelets.^51^ For patients taking warfarin, it is advised that INR is checked on admission and 5 mg IV vitamin K is administered early while the patient is in the Emergency Department. However, if the patient is deemed to be at high risk of thrombosis, the guidelines recommend seeking advice from haematology services. Similar to the BCSH guidelines, the threshold INR level for proceeding to surgery has been set at ⩽1.5. INR is advised to be rechecked at 6 am on the day of surgery and if necessary, a further 2 mg IV vitamin K dose should be administered and INR rechecked again 4 hours later. Where it is believed that residual anticoagulation threatens possibility of surgery that day then the guidelines advise use of PCC. This may also be used if surgery on the day of admission is feasible however warfarin anticoagulation has not been reversed to the threshold level. The use of FFP is explicitly discouraged, however. Regarding the management of patients taking DOAC medications, recommendations are similar to the BCSH guidelines where timing of surgery is based on the specific anticoagulant, bleeding risk, and renal function. For patients taking dabigatran and surgery is predicted to be delayed more than 24–48 hours, Idarucizumab may be given after discussing with the haematologist. In terms of neuraxial anaesthesia, this is advised when INR level is <1.5 in the case of warfarinsed patients, and only when DOAC effect can be excluded such as by anti-Xa assay results. Similar to the BCSH guidelines, tranexamic acid (1 gram IV) is advised to be considered for all patients except those with disseminated intravascular coagulation. For patients taking aspirin or clopidogrel, the guidelines advise proceeding promptly with surgery unless the patient is taking the latter and deemed high risk of bleeding, in which case it should be withheld for 24 hours preoperatively.

## Comparison of perioperative management

Several strategies involving varying interventions for the management of anticoagulated patients have been published at a local and national level. This has likely contributed to the wide variation in clinical practice of managing these patients. Alcock et al.^55^ in their recent meta-analysis compared 4 possible options in approaching the management of DOAC anticoagulated femoral fracture patients including plasma product reversal, antidote reversal, time-reversal, and non-reversal. The latter 2 were defined as surgery beyond and within 36 hours of presentation respectively, in line with national guidance. Only 1 study was identified which performed a direct comparison between time-reversal and non-time reversal, categorised using a time to surgery cut-off of 24 hours (2/51 versus 0/40 deaths respectively; OR 4.09; 95% CI, 0.19–87.65).^56^ For the remainder interventions, a network meta-analysis was constructed due to a lack of studies performing direct comparisons between treatments using the authors criteria. Using this approach, no differences were observed in mortality when comparing “time reversed” versus “non-time reversed” DOAC patients (OR 1.48; 95% CI, 0.29–7.53 and OR 1.63; 95% CI 0.56–4.76). However, crude rate of mortality reported in these studies was found to be almost double in the “time reversed” patient group (5.14% of 194 vs. 2.88% of 208 patients). Indirect comparison of blood transfusion requirements also revealed no differences between groups (OR 1.16; 95% CI, 0.42–3.23 and OR 1.61; 95% CI, 0.76–3.40). Although no statistical analyses were possible to be performed for outcomes hospital length of stay and time to surgery, crude average mean/median times for these were 15.2 days and 45.6 hours (95% CI, 44.3–47.0) in the “time reversed” group and 8.5 days and 24.8 hours (95% CI, 24.0–25.6) in the “non time-reversed” groups respectively. The review authors concluded that although they were unable to exclude harm or benefit, operating within 36 hours appeared to be safe.

A similar study comparing the outcomes of different possible management strategies in warfarinised patients does not seem to exist and is a gap in the literature worth addressing. However, existing studies have shown that administration of vitamin K is effective and relatively safer than other therapy strategies involving use of FFP and PCC.^39,41,42,57^ It is important to emphasise that warfarin has a long half-life of 35 hours and employing a “time reversal” strategy would unlikely permit expedited surgery.^32^ Furthermore, the half-life of vitamin K is relatively shorter at approximately 25 hours, which means single dosing may not be sufficient. Modelling and simulation techniques have been performed in an attempt to determine the optimum vitamin K reversal strategy in hip fracture patients. Results of this study revealed substantial variability in pharmacokinetic and pharmacodynamic profiles of patients for both warfarin and vitamin K. Only half of the studied population were believed to have favourable kinetics and these patients needed a minimum of 20 hours from administration of 5 mg IV vitamin K to enable their INR level to fall below 1.5 emphasising the urgent need for early reversal. However, single dosing was not sufficient in the remaining half of patients to enable a similar rate of reversal of INR.^57^ These findings closely resemble the protocol of Diament et al.^37^ recommending an initial administration of 2 mg IV vitamin K prior to blood testing with further subsequent doses based on the INR result.

## Timing of surgery and perioperative outcomes

Many studies investigating blood loss and transfusion requirements in anticoagulated femoral fracture patients undergoing early surgery have demonstrated no significantly increased risk of these events compared to non-anticoagulated patients. In a recently published retrospective cohort study consisting of 41 patients taking DOAC medications and 494 non-anticoagulated patients who all underwent surgery at approximately 20 hours following admission, there were no significant differences in transfusion requirements, major bleeding, and 30-day mortality. Interestingly, there was a significant reduction in the difference between pre- and postoperative haemoglobin levels in the non-anticoagulated patient group (1.0 vs. 1.8 g/dL, *p* < 0.01).^58^ This may be due to more meticulous haemostasis being achieved by the operating surgeon intra-operatively due to the belief that DOAC patients have an increased risk of bleeding. All these findings were despite patients taking DOAC medications being slightly older (81.7 vs. 77 years; *p* = 0.02) and having a higher body mass index (BMI) (26.9 vs. 24.2 kg/m^2^; *p* = 0.01) which are both associated with reduced resting haemoglobin levels.^59^ The latter is also associated with an increased bleeding risk.^60^ Subgroup analyses based on type of surgical procedure and logistic regression analyses were performed to adjust for preoperative haemoglobin differences. These also revealed no significant increases in blood loss or transfusion requirements in patients taking DOAC medications. There were also no differences in deep infection rates or need for further surgery for seroma or haematoma complications between patient groups.

Similar findings were demonstrated in a different retrospective multicentre study by Franklin et al.^61^ who also found no differences in change in haemoglobin levels, transfusion rates, wound complications, re-operation, and survival up to 1 year when patients anticoagulated with DOAC medications underwent surgery at an average 28.9 (standard deviation [SD] 11.8) hours from admission compared to 21.4 hours (SD 12.4) in non-anticoagulated patients. However, the anticoagulated patient group were readmitted at a significantly higher rate compared to the control group (21% vs. 5.3%; *p* = 0.05) although these were all unrelated to the surgical site. Rather, these were mostly due to cardiac and cerebrovascular events. These are likely related to cessation of their DOAC medication on admission and perhaps further supports the need for expedited surgery in helping to limit the duration of time this patient group is restricted from taking their anticoagulant medication.

In addition to these reasons for readmission, other contributing causes for these patients’ relatively higher mortality could be associated with delayed surgery and increased risks of recumbency related complications including pneumonia, urinary tract infections, sarcopenia, and decubitus ulcers.^62–65^ These patients are perhaps more susceptible to experiencing these complications due to their relatively greater number of comorbidities. For these reasons, prioritising earlier surgery or more frequent orthogeriatrician review may improve outcomes in this patient group.^66^

Schermann et al.^66^ compared outcomes including 1-year mortality following closed reduction internal fixation and hip hemiarthroplasty among patients taking DOAC medications versus those not taking any anticoagulants. Their multivariate logistic regression demonstrated that mortality risk was similar between the 2 patient groups however surgical delay itself was an independent predictor of 1 year mortality. Similar analyses for blood transfusion found no significantly different results for the use of DOAC medications.

Another multicentre study by Levack et al.^67^ utilised propensity score matching on multiple variables including institution, age, sex, year of surgery, type of surgery, and comorbidities. In a subgroup analysis comparing DOAC patients undergoing surgery within (*n* = 37) and after (*n* = 95) 24 hours, results revealed no differences in transfusion rates (*p* = 0.558) or overall complication rates (*p* = 0.179) between these 2 groups. Multivariable logistic regression analyses involving the entire patient cohort (*n* = 393) and controlling for age, sex, BMI, time to surgery, and type of surgery revealed that DOAC use was neither predictive of blood transfusion (OR 0.83; 95% CI, 0.47–1.5, *p* = 0.536) or a composite complications outcome (OR 1.5, 95% CI, 0.86–2.7; *p* = 0.150).

Similar studies have been carried out in patients taking warfarin medications. Levack et al.^68^ carried out another study utilising propensity score matching and including the same variables mentioned above. They found a slightly higher transfusion rate amongst warfarinised patients compared to non-anticoagulated patients during admission (52.4% vs. 43.3%; *p* = 0.032) although median number of units transfused were similar between groups (2 units, *p* = 0.456). In contrast to previous studies,^14,46^ this observed difference was driven by arthroplasty (50% vs. 28.5%; *p* = 0.001) rather than cephalomedullary nailing procedures (61.8% vs. 62.5%). Although tranexamic acid was administered to <5% of patients in both groups, the rate of blood transfusions remains higher compared to other studies in which tranexamic acid was not given.^13^ A possible explanation may be the lack of a standardised transfusion protocol resulting in these differences between groups. Haemoglobin <70 mg/dL comprised 19% versus 32% of reasons for transfusions in warfarinised and non-anticoagulated patients respectively. Interestingly, a sub analysis of warfarinised patients by INR thresholds of 1.5, 1.7, and 2.0 showed no differences in overall transfusion rates (*p* = 0.092), 90-day readmissions (*p* = 0.31), complications (*p* = 0.999), or postoperative mortality (*p* = 0.571). However, when considering all patients, the rate of 90-day readmission was over 3-fold greater compared to non-anticoagulated patients (31.4% vs. 8.9%; *p* = 0.001). Among the reasons cited, significant differences between groups were found for acute kidney injury and anaemia only. 90-day complications were also significantly higher in the warfarinised group (46.7% vs. 38.1%; *p* = 0.039) with significant differences between groups for pneumonia only.^69^ This may be attributed to delayed surgery and could also have influenced the rate of readmission for acute kidney injury.^62,63,69^ Multivariable logistic regression showed that warfarin use, and INR level on admission and day of surgery were not independent predictors for transfusion or complications.

In a different comparative cohort study involving 124 femoral fracture patients, surgery was carried out for the warfarinised patient group with INR values ranging from 1.0 to 3.1 on the day of their procedure.^20^ Patients were closely matched on age, sex, type of surgery and year of surgery. A sub analysis comparing outcomes in warfarinised patients with INR <1.5 (*n* = 41) to ⩾1.5 (*n* = 21) was performed. Mean INR values and time to surgery from presentation for these groups were 1.25 and 1.77 (*p* < 0.01), and 54 and 33.3 hours (*p* < 0.01), respectively. However, there were no significant differences in transfusion rates (63.4% vs. 47.6%), calculated blood loss (1246 ml versus 1082 ml), complications (26.8% vs. 19%), readmissions (9.8% vs. 19%) and in-patient mortality (2.4% vs. 9.5%) between the 2 patient groups for this sub analysis, respectively. Unsurprisingly, general anaesthesia was performed in all patients with INR ⩾ 1.5 compared with 39% of patients with INR < 1.5. Multivariate logistic regression modelling, controlling for antiplatelet use and type of surgery, determined that day of surgery INR was not associated with blood transfusion (OR 0.33; 95% CI. 0.10–1.13, *p* = 0.078). Rather, cephalomedullary nailing was the only covariate identified which was associated with the latter (OR 3.3; 95% CI, 1.01–10.74; *p* = 0.05) echoing findings of previous studies.^46^ This paper suggests that operating at an INR value of 1.8 which was the mean value for differentiating patient subgroups used in the analyses can help to significantly reduce time to surgery from presentation without increasing the risk of complications.

Another similar study by Kain et al.^70^ compared postoperative complications between 216 warfarinised patients grouped into low (<1.5) and high (1.5–3.0) INR levels at time of surgery. Both time to surgery from presentation and length of stay were significantly reduced in the high INR group (1.21 vs. 1.86 days; *p* = 0.006, and 6.46 vs. 8.26 days; *p* = 0.008 respectively). Although anaemia was not defined in the study, the authors report that this was more common in the high INR group (22.8% vs. 10%; *p* = 0.02). However, there were no significant differences for transfusion requirements and estimated surgical blood loss; 25% versus 41% (*p* = 0.61) and 241 ml versus 195 ml (*p* = 0.78) respectively. These slightly conflicting findings may be related to a lack of a standardised blood transfusion protocol. When considering all causes for reoperations and complications in the study by Kain et al.^70^ there were no significant differences demonstrated between the groups for these outcomes (*p* = 0.89 and *p* = 0.12 respectively). However, it may be worth mentioning that 2 patients in the high INR group experienced haematomas which occurred following a hip hemiarthroplasty and cephalomedullary nailing procedure. The patient who underwent the former procedure had an INR of 1.7 and their haematoma was managed non-operatively although required antibiotics at 6 weeks postoperatively for a superficial wound infection. However, the latter patient who had an INR of 2.3 required re-operation for haematoma evacuation. Nonetheless, and for perspective, these events are substantially fewer than the 15% incidence of reported haematomas in a different large study of unselected patients undergoing hip hemiarthroplasty.^71^ The authors also investigated 30-day mortality and found no differences between patient groups.

A recently published meta-analysis investigated outcomes between femoral fragility fracture patients taking aspirin and/or clopidogrel compared to those not using either medications.^72^ The authors categorised patients as having early and delayed surgery using a cut-off time from admission to theatre of 5 days, and included a total of 9 studies which met this definition. The findings of this study demonstrated that patients who underwent early surgery had a relatively greater change in the difference in haemoglobin values (weighted mean difference 0.75, 95% CI, 0.50–1.00, *p* < 0.001) however there were no differences in the number of blood transfusion events (OR 0.99; 95% CI, 0.55–1.77; *p* = 0.97) and mean number of units transfused (mean difference 0.16, 95% CI, -0.28–0.59, *p* = 0.47). Although there were no differences in 30-day mortality between groups (OR 0.53; 95% CI, 0.15–1.95; *p* = 0.34), early surgery conferred a protective effect at 3 months postoperatively (OR 0.36; 95% CI, 0.14–0.97; *p* = 0.04). However, this difference was no longer present at 1 year postoperatively (OR 0.48; 95% CI, 0.16–1.41; *p* = 0.18).

## Discussion

Management of the anticoagulated fragility femur fracture patient is a contentious topic and there is varied practice in hospitals around the world. Despite being 1 of the most common injuries and patient groups, there is substantial variation in the management process in many areas,^10,73,74^ and total absence of specifics when related to fractures outside of the proximal femur. There is stark evidence of both inter-hospital variation in the management of anticoagulated patients as well as disparity compared to non-anticoagulated patients. Nonetheless, it is somewhat reassuring that the evidence suggests increased development and reliance on protocols and guidelines which have helped to improve outcomes in this patient group.^38^ However, further progress is needed including development of updated and more specific guidance focussing specifically on this topic and incorporating the most recent evidence to help reduce variation in management and ensure outcomes for these patients continue to improve over time.^75^ The ‘fragility femur fracture patient’ is now recognised across databases as having similar need overall to that which was previously confined to the hip yet we could find no evidence to guide overall care.

Standardisation of the management of fragility femur fracture patients in general has helped improve outcomes over time, and a similar approach for the subgroup of patients taking anticoagulants and antiplatelets requires adopting.^76^ A survey conducted in Canada revealed that almost 3 in 4 responding orthopaedic surgeons did not believe adequate clinical guidelines for this topic existed. Furthermore, less than 1 in 4 surgeons reported carrying out expedited surgery on patients taking DOAC medications despite compelling evidence to do so.^73^ In the UK, the full scale of the issue is currently being investigated through the Hip and femoral fracture Anticoagulation Surgical Timing Evaluation (HASTE) project.^77^ It is important that changes in management are implemented as early as possible to consider not just current circumstances but account for the projected future population of fragility femur fracture patients of whom a relatively greater proportion will be taking anticoagulant and antiplatelet medications.

Focus also should be on the projected rise in the relative incidence of periprosthetic femoral fragility fractures due to an ageing population and the increased demand for arthroplasty procedures.^78–80^ Compared to procedures for hip fractures and native bone femoral fractures, fewer surgeons perform procedures for periprosthetic femoral fractures which may affect the process of expedited surgery for this patient subgroup. This is an additional challenge to be considered for the future planning of the healthcare service.^6^ Management of patients sustaining these injuries as well as native bone femoral fractures are now audited on a national level owing to the recent expansion of these patient groups as part of the National Hip Fracture Database (NHFD) of England and Wales.

Throughout this review we have generalised the findings from studies focussing on the hip fracture patient population to the broader population of individuals with fragility femur fractures. This may be a limitation of this review, however, these patient groups share numerous commonalities, such as comparable levels of frailty and comorbidity, susceptibility to medical and surgical complications, as well as indications for oral anticoagulation and antiplatelet medications.^4,5,62–65^

## Conclusion

Expedited surgery within 36 hours in patients taking VKAs, DOACs, and antiplatelets is feasible and safe. However, many surgical teams still delay patients unnecessarily. For patients on warfarin, early intravenous administration of vitamin K while the patient is in the Emergency Department and prior to return of blood tests results is effective in reducing INR to levels most surgeons would be comfortable with.^20,26,37^ Whilst some guidelines have suggested use of FFP and PCC for reversal, these carry substantially higher risks compared to vitamin K and their use is not typically necessary thus we feel, based on our evidence, should be avoided.^42^ For patients taking DOACs and antiplatelets, there is little benefit in delaying surgery. Despite residual anticoagulation effects, the risk of morbidity and mortality is most likely greater when surgery is delayed in this patient group. Also, increasing the duration of time that the anticoagulant medication is withheld from the patient may increase their risk of medical complications.

An evidence base previously confined to the proximal femur is still not fully embraced and utilised by the surgical community. Patients still are needlessly delayed. Following this review and awaiting the publication of the findings of the HASTE study, it is fundamental that a consensus statement co-badged and contributed to by the relevant advisory bodies in haematology, anaesthesia, older peoples medicine and surgery is produced and embraced. This will help address the current situation of widely varied practice and patient delays.
